# Prevention of early complications following total hip replacement

**DOI:** 10.1051/sicotj/2021060

**Published:** 2021-11-30

**Authors:** Andreas Fontalis, Daniel J. Berry, Andrew Shimmin, Pablo A. Slullitel, Martin A. Buttaro, Cao Li, Henrik Malchau, Fares S. Haddad

**Affiliations:** 1 Department of Trauma and Orthopaedic Surgery, University College London Hospitals London NW1 2BU UK; 2 Mayo Clinic Rochester MN 55905 USA; 3 Melbourne Orthopaedic Group Windsor Victoria 3181 Australia; 4 Monash University Windsor Ontario N9B 3P4 Australia; 5 Institute of Orthopaedics “Carlos E. Ottolenghi”, Italian Hospital of Buenos Aires Buenos Aires Argentina; 6 Department of Orthopaedics, First Affiliated Hospital of Xinjiang Medical University 137 South LiYuShan Road Urumqi Xinjiang 830054 China; 7 Harvard Medical School, Harvard University Boston MA 02115 USA; 8 University College London, Division of Surgery and Interventional Science Gower Street London WC1E 6BT UK

**Keywords:** Hip Arthroplasty, Early complications, Prevention strategies, Education, Risk stratification

## Abstract

Total hip arthroplasty (THA) has been quoted as “the operation of the century”, owing to its efficacy and the substantial improvements evidenced with respect to functional patient outcomes and quality of life. However, early postoperative complications are often inevitable, hence it is imperative to take every step to prevent them and minimise morbidity and mortality. This manuscript focuses on the most common early complications following THA, namely venous thromboembolism (VTE), prosthetic joint infection, periprosthetic fracture, instability, and leg length inequality. It aims to outline effective risk stratification strategies and prevention measures that could apply to the wider Orthopaedic community.

## Introduction

Attempts at replacing the hip joint have been recorded as early as 1891 in Germany, while the late 19th and early 20th century witnessed surgeons experimenting with the interpositional arthroplasty. However, it was not until 1953 when George McKee adopted a metal-on-metal articulation, using the modified Thompson stem [1]. Later on, Sir John Charnley, who is considered the father of modern Total hip arthroplasty (THA), spearheaded the efforts towards developing the so-called “low friction arthroplasty” that shares similar principles to the prostheses used today; consisting of an acetabular component, a polyethylene component and a metal femoral prosthesis [1].

Total hip arthroplasty (THA) is a highly successful and cost-efficient procedure that has been shown to substantially improve functional pain outcomes and quality of life. According to the latest National Joint Registry Report, over 100,000 THAs have performed annually in the UK, whereas the respective number in the United States is greater than 370,000 [2, 3]. Owing to the ageing population and increased longevity, the demand for THA is growing exponentially and is projected to increase to 635,000 procedures by 2030 in the USA alone [3].

Most complications, whether procedure-specific or systemic, are infrequent and can be effectively prevented by employing prevention strategies. Venous thromboembolism (VTE) is one of the commonest preventable complications following THA and carries a high mortality risk. The aetiology is multifactorial, and the involvement of several factors has been suggested, including stasis secondary to the manoeuvres performed during THA, long bone reaming and intimal injury. In a large meta-analysis among 21,369 patients receiving chemoprophylaxis after THA or hemiarthroplasty, authors reported that the pooled rates of symptomatic VTE before discharge were 0.53% (95% CI, 0.35%–0.70%), while the pooled rates for symptomatic deep vein thrombosis (DVT) and pulmonary embolism (PE) were 0.26% (95% CI, 0.14%–0. 37%) and 0.14% (95% CI, 0.07%–0.21%) respectively [4]. In another large retrospective cohort study encapsulating 26,391 patients undergoing primary or revision THA over 10 years, the reported risks of postoperative symptomatic PE and fatal PE were 1.1% and 0.02% [5].

Prosthetic joint infection (PJI) is a devastating complication following THA, with far-reaching implications for the patient and healthcare resource utilisation. The reported incidence of deep PJI for primary THA is approximately 1% [6]. Management of PJI is extremely challenging and involves multiple interventions, prolonged administration of antibiotics considerably impacting patients’ quality of life while the economic burden in the UK for an infected THA is estimated at a mean of £50,000 for a revision procedure [6]. Considering the projected increase in the demand for arthroplasty, it becomes clear that effective, evidence-based prevention measures are vital to prevent a concurrent increase in the prevalence of PJI.

Furthermore, it is very important to prevent avoidable intra-operative complications that lead to poor patient outcomes and dissatisfaction. The majority of intraoperative femoral fractures occur during stem insertion, and the reported incidence in the literature ranges between 0.1% and 1% with the use of cemented implants and 3%–18% with uncemented [7, 8]. Several factors have been associated with a higher risk of periprosthetic fracture, including osteoporosis, inflammatory arthropathy, age, and the use of cementless implants [7].

Another commonly encountered, avoidable sequela following THA is leg-length inequality (LLI). The reported incidence of LLI in the literature following primary THA ranges from 1% to 27%, while the mean length discrepancy varies from 3 to 17 mm [9]. Care must be taken with respect to risk stratification and appropriate patient counselling for high-risk groups. Patients’ perception of leg-length inequality should also be documented as it has been reported that a significant inequality of as much as 20 mm is often evident in otherwise asymptomatic individuals [10, 11]. Despite the lack of evidence, a discrepancy of less than 10 mm has been reported to be acceptable [10], whereas patients with underlying spinal pathology or substantial systemic comorbidity (pulmonary, cardiac, neuromuscular) are less likely to tolerate leg length inequality [11].

Finally, dislocation constitutes the most common indication for early revision following primary THA [7]. A recent study encompassing 16,186 THAs reported the adjusted risk of instability within 2 years ranged between 0.17% and 1.74% with different approaches [12]. The influence of the surgical approach has also been a focus of debate; several reports have suggested a higher dislocation rate with the posterolateral approach; however, performing a posterior capsular repair has been shown to reduce dislocation to less than 1% [13]. Other factors that should be considered involve orientation of the cup and femoral components, abductor deficiency, spinopelvic pathology and the choice of articulation based on pre-operative risk stratification.

The International Hip Society partnered with SICOT PIONEER to present its Webinar on strategies to prevent early complications following THA. The following review summarises the presentations from thought leaders on the subject and illustrates prevention strategies that can apply to the wider Orthopaedic community.

## Thromboprophylaxis in primary total hip arthroplasty

It has been shown that total hip arthroplasty (THA) is mechanically pro-thrombotic since femoral vein occlusion does naturally exist when combining maximum flexion with either internal or external rotation [14]. Venous thromboembolism (VTE) has therefore been a matter of concern since the early history of THA, mostly with the introduction of cemented stems. A terrible triad of factors, including aggressive femoral canal preparation, use of cement, and maximum lower extremity rotation, led Huo et al. and Sharrock et al. to recommend routine use of intraoperative unfractionated heparin after insertion of the cup [15, 16].

A link between genetic predisposition and developing VTE has been assumed, especially in non-O blood group individuals [17]. Notwithstanding, a percentage between 35% and 84% will suffer a thromboembolic event without any prophylaxis after undergoing primary THA, and 0.5%–4% might even develop one despite administering a chemoprophylactic agent [18]. VTE should therefore be considered to have a multifactorial origin, and prophylaxis should be multimodal [19].

Undeniably, prophylaxis commences in the operating room, using spinal hypotensive anaesthesia, given the higher complication rate (including more thromboembolic events) when general anaesthesia is employed [20]. Tranexamic acid has been proven safe and efficacious in both primary and revision THA [21], whenever there are no absolute contraindications [22, 23]. It has also resulted in dramatically lowering the rate of blood transfusions, hence orthopaedic surgeons should routinely use it to prevent haemorrhagic complications [24]. It has been acknowledged that prophylaxis should be multimodal [25]. This includes discontinuation of procoagulant medications, regional anaesthesia, rapid mobilisation and the use of compression stockings and devices, and postoperative chemoprophylaxis tailored to the patient’s risk. Besides performing the procedure in a timely fashion and beginning to bear weight and mobilisation as soon as possible, chemoprophylaxis plays a key role in preventing VTE.

Consequently, risk stratification is instrumental with respect to decision making. Specific criteria to define “high-risk” patients, which will benefit from a more “aggressive” prophylactic agent, include: greater than Class I heart failure as per the New York Heart Association (NYHA) Functional Classification system; prior VTE within the last 5 years; atrial fibrillation treated with warfarin or similar agents; thrombophilia or hypercoagulable state; active cancer; morbid obesity (body mass index [BMI] >40) [26]; and age >70 years old [27, 28]. While low-risk patients can be safely covered with aspirin, higher-risk patients will need enoxaparin or warfarin, besides a pneumatic compression device. With this approach, the incidence of VTE can be reduced to less than 1% in both low- and high-risk patients.

Following the results of large, well-designed clinical trials [29–31], aspirin has gained popularity in THA prophylaxis as it is inexpensive, easy to administer, reasonably well-tolerated safe, and there is no need for monitoring. Although several regimens have been described, the current evidence shows that doses of low-dose (i.e. 81–100 mg/daily) can be as effective as higher [32]. A recent randomised controlled trial comparing rivaroxaban alone (10 mg once daily starting on postoperative day (POD) 1 for a total of 14 days) with the “Wells Protocol” (rivaroxaban 10 mg once daily from POD 1 up to and including POD 5, plus aspirin 81 mg once daily for 30 days starting on POD 6) showed the latter was non-inferior (*p* = 0.01). Superiority for the prevention of VTE could not be established (*p* = 0.84), and no difference was evident regarding bleeding events [33]. This protocol has also proven to be safe and effective in the context of outpatient THA [34].

Direct thrombin inhibitors (oral dabigatran 220 mg once daily or 150 mg once-daily if renal failure is present for 30 days) and Xa inhibitors (apixaban 2.5 mg orally twice daily for 35 days) have also been recommended for VTE prevention for low-and high-risk patients [35, 36]. When compared to enoxaparin, both classes have proven to be superior in decreasing VTE events without increasing the bleeding risk. None of these agents has been associated with an increased risk of surgical site infection. A recent meta-analysis showed that warfarin used for prophylaxis in THA was associated with a higher risk of deep infection (OR = 1.929, 95%CI 1.197–3.109, *p* = 0.007) compared with aspirin. Furthermore, results did not unveil an increase in the infection risk when comparing warfarin to heparin and heparin to aspirin [37].

Table 1 depicts our institutional guidelines for VTE prophylaxis in patients undergoing THA based on risk stratification.

**Table 1:** Institutional protocol for VTE prophylaxis in patients undergoing THA based on risk stratification.

Risk stratification	Regimen	Duration
Low-risk patients (planned for fast-track or outpatient THA)	Rivaroxaban 10 mg and aspirin 100 mg	Rivaroxaban 10 mg once daily for 5 days starting on POD 1 plus aspirin 100 mg once daily for 30 days
Intermediate-risk patients (older than 60 years old, bilateral THA, diabetic, greater than Class I heart failure, atrial fibrillation, or varicose veins)	Enoxaparin 40 mg + Dabigatran 220 mg/d (or 150 mg/d, adjusted for weight and renal function)	Enoxaparin 40 mg starting on POD 1, dabigatran 220 mg/d (or 150 mg/d adjusted for weight and renal function) for 30 days commencing upon discharge [38]
High-risk patients (history of VTE, active cancer, greater than Class I congestive heart failure or atrial fibrillation treated with anticoagulant, thrombophilia, prolonged immobility, BMI >40, steroid administration, hip fracture)	Enoxaparin 40 mg	Enoxaparin 40 mg once daily starting on POD 1 for 30 days.

POD: Postoperative Day.

## Preventing prosthetic joint infection

Prosthetic joint infections (PJI) after total hip arthroplasty (THA) are a devastating complication. Not only does it increase morbidity, but it also constitutes a significant burden for healthcare systems. The incidence of infection following primary THA will steadily increase as the demand for THA is progressively rising globally [39]. The most challenging aspect in preventing and treating PJI is the implant-related biofilm [40], as the number of bacteria needed to induce infection is 1000 times lower in the presence of an implant [41]. Prevention strategies should be employed in all stages, before- during- and after the surgical intervention.

Table 2 illustrates pre-operative risk factors. Weight optimisation is key for patients with obesity or malnutrition status. There are several reports in the literature highlighting the association between tobacco use and increased risk of PJI. In one of the largest prospective studies encompassing 8559 total joint replacements, smoking resulted in a 1.8 fold risk of prosthetic joint infection [51]. In concordance, a recent systematic review and meta-analysis including 10 studies and 20,640 patients corroborated the association of PJI with tobacco use [52]. Hence it is recommended that smoking should stop at least four weeks before surgery [42], and THA should be performed at least three months after an intra-articular injection [53]. Pre-operative aspiration and microbiology input regarding antibiotic administration is required for patients with a history of septic arthropathy. The optimal interim period for performing a THA after septic arthritis is debatable, and no consensus has been reached [54]. A recent multicentre study employing a receiver operating characteristic (ROC) analysis indicated that 5.9 months after the initial treatment might represent a suitable timepoint. However, when the population was dichotomised by this threshold, the authors reported no difference with respect to the PJI rate [55].

**Table 2:** Pre-operative risk factors for PJI.

Diabetes mellitus	HbA1c > 7%
	Fingertip blood glucose >200 mg/dL [42]
Obesity	BMI > 35 kg/m^2^ [26, 43, 44]
Malnutrition	Serum albumin < 3.5 g/dL [42]
Renal insufficiency [45]	Serum creatinine >1.5 mg/dL; Creatinine clearance rate < 100 mL/min
Anaemia	(Hb < 9 g/dL) [46]
Smoking	
Recent history of intra-articular injection [47]	
Inflammatory arthritis [48], Previous septic arthropathy [49]	
Systemic infectious process [50]	

Intra-operative modifiable factors include Operating Room (OR) environment, surgical attire, surgical site preparation, surgical technique, wound management, antibiotic prophylaxis and blood loss. The effect of laminar airflow (LAF) on reducing the incidence of PJI is debatable, and vertical LAF is preferable [42]. Table 3 summarises intra-operative measures and strategies for PJI prevention.

**Table 3:** Intra-operative measures and strategies for preventing PJI.

Measures	Strategy
Limit OR traffic	Ensuring access to frequently used instruments, utilisation of intercom, and keep door movements per surgical procedure to a minimum
Double gloving	Gloves should also be changed every 30–60 min [56]
Meticulous skin preparation	Iodine and alcohol-based solutions are recommended for skin preparation [57]. Diluted betadine is recommended by WHO and CDC [57]
Frequent change of blade	Blade change following skin incision
Reduce operative time	Prolonged operative time increases the risk of wound contamination [58]
Thorough irrigation	Pulsed lavage efficacy of eradicating organisms by 100 times greater than that of a spherical syringe [59]
Prophylactic administration of antibiotics	A first-generation cephalosporin [60] or clindamycin should be administrated within 30 minutes of the incision.Vancomycin should be considered for *Methicillin-resistant Staphylococcus aureus* (MRSA) colonised patients or patients with a previous MRSA infection.
Minimise blood loss	Tranexamic acid is recommended for all THA patients unless there is a significant risk of embolism [61].

Aggressive chemical anticoagulation postoperatively should be avoided, and early mobilisation is recommended [62]. Many studies have shown aspirin to be a safe and effective agent for VTE prophylaxis with a favourable risk profile [63].

## Prevention of periprosthetic femur fractures during THA

Periprosthetic femur fractures have become one of the most common complications of THA as other complications have declined and as the pool of patients with THA increases. Intraoperative and early postoperative fractures may be reduced in frequency with several effective prevention strategies [64]. Intraoperative fractures may be reduced by using cemented femoral implants in “high risk” patients, especially those elderly with poor bone [65]. If uncemented implants are used, periprosthetic femur fractures may be reduced by careful pre-operative templating to optimise implant choice based on implant-fit with individual patient anatomy; surgeon care during broaching and implant seating to avoid excessive force; and the use of prophylactic wire or cable cerclage in “at risk” patients. Early postoperative fractures are especially common after uncemented tapered stem insertion (rates in many studies are 0.5% to 1% [66]); these may be prevented by: selected use of cemented stems in “at risk” patients; and prophylactic femoral cerclage of “at risk” patients when tapered uncemented stems are chosen [65]. When using cemented stems, surgeons should be aware that polished collarless “taper slip” type implants have a higher risk of postoperative periprosthetic femur fractures than collared “shape-closed” [67–70] or composite beam-type stems. In complex primary THA, several strategies help reduce periprosthetic femur fractures: (1) in patients with retained hardware that requires removal, a sequence of (a) hip exposure, (b) hip dislocation, (c) hip relocation, (c) hardware removal, (e) hip re-dislocation reduces the risk of spiral fractures during the initial hip dislocation when torsional forces that can lead to fracture are highest; (2) prophylactic cerclage of metaphyseal or diaphyseal stress risers from previous hardware or bone defects. For revision THA, periprosthetic femur fractures may be prevented by judicious use of extended greater trochanteric osteotomy for implant removal and bypassing areas of weakness in the femur with longer revision implants when appropriate.

With the popularity of minimally invasive approaches, such as the Direct Anterior Approach (DAA), have gained over the recent years, it is imperative to explore the safety and patient outcomes following minimally invasive strategies. There is mounting evidence to suggest that DAA is associated with longer operating theatre times [71], albeit an improvement has been noted when arthroplasty surgeons move past the DAA learning curve. Owing to the substantial incongruity of published studies regarding the association of DAA with intra-operative periprosthetic fractures, no safe conclusions can be made [72]. Early reports suggested a higher incidence of periprosthetic fractures, while a recent population-based study encompassing 5986 propensity-score matched patients found a statistically significant increase of experiencing a major complication following DAA [73]. Notwithstanding this, other recent publications [74] and quantitative synthesis of existing evidence have failed to unveil such a relationship [75].

In summary, the main strategies to prevent periprosthetic femur fractures are selected use of cemented stem fixation; liberal use of prophylactic cerclage; thoughtful, sequential exposure in high-risk patients; and protecting/bypassing major stress risers.

## Prevention of instability

Joint Replacement Registry data and large institutional reviews indicate that dislocation, fracture and leg length discrepancy still occur as complications of total hip replacements. Improving outcomes in total hip replacement in the future will come from reducing these often avoidable complications.

To prevent episodes of instability, we must understand the aetiology.

Aetiology can be discussed under the headings of Surgical Procedure Factors, Patient Factors and Implant Factors.

### Surgical procedure factors

1. Acetabular component positioning [76], most notably achieving the patient-specific cup position, not the generic Lewinnek’s safe zone.
2. Stem positioning must initially be set out to reproduce the native version as this is the current standard of care, but in some situations, impingement can be avoided by using modular femoral components to change the version to minimise impingement.
3. Getting correct soft tissue tension by optimising offset and leg length.
4. The surgical removal of osteophytes to avoid bony impingement.

### Patient factors

Patient factors that need to be considered include:

1. Spino-pelvic pathology that can affect functional cup position and the risk of impingement [77, 78]:
  - Adverse sagittal pelvic mobility [79];
  - Spinal imbalance [80];
  - Spinal stiffness is often associated with prior spinal fusion surgery.
  A recent presentation at the American Association of Hip and Knee Surgeons (AAHKS 2020) annual meeting suggested that 80% of a group of total hip replacements that were revised for instability had one or more of the above spinopelvic risk factors.
2. Pre-operative or intraoperative diagnosis of abductor deficiency.
3. Known neurological conditions, for example, Parkinson’s disease
4. Primary diagnosis of fractured neck of femur or Avascular Necrosis

### Implant factors

Implant-related factors can also influence the occurrence of instability.

1. The use of modular femoral components where the stem version can be changed needs to be considered in some situations to avoid implant on implant impingement.
2. Increasing head size and the use of Dual Mobility articulations [81, 82] can also contribute to a reduction in instability [83] in those who are known to be at risk [84].
3. Constrained acetabular liners are now mainly used for revision scenarios but can be considered in very high-risk patients.

In summary, to reduce the incidence of instability in THA, we must understand the potential aetiologies, identify the at-risk patient, plan patient-specific orientation of cup and femoral components and choose an articulation based on pre-operative risk assessment.

## Prevention of leg length inequality

Leg length inequality is an avoidable problem following total hip arthroplasty that can lead to a great deal of patient dissatisfaction and is an independent risk factor for poor outcome [85] and litigation.

Whilst it is impossible to always get leg length completely equal, and surgeons often have to compromise length and offset to manage with the implants available and gain stability, it is critical that every effort is made to minimise the problem. The best way to do so is to plan carefully by identifying high-risk patients, templating accurately, and having multiple cross checks to avoid inadvertent lengthening.

It is always important to identify at-risk patients. These are particularly those with atypical bony anatomy where the socket may be inserted at the wrong level or the stem may end up sitting proud or those who are less likely to cope with leg length inequality such as individuals of short stature, those who already have a shortened contralateral limb, those who have scoliosis or those who have fixed contractures.

It is beholden upon the surgeon to carefully examine the patient preoperatively, assess the patient’s perception of leg length inequality and identify any fixed deformities. If in doubt, appropriate imaging such as standing radiographs and formal assessment of the lumbar spine and spinopelvic relationship [78, 79] should be performed.

On the table, it is important for the surgeon to understand both the leg length inequality the patient already has and the aim of the procedure in terms of leg length and, if so, what correction is required.

It is important to have a target position in mind relative to easily identifiable landmarks to work intraoperatively, including the teardrop, the true floor and the superior lip of the acetabulum, and the lesser and greater trochanters. This allows the surgeon to check intraoperatively as to whether the pre-operative plan is being achieved. Centre of rotation is another extremely good marker which we frequently use alongside comparing the resected head with the trial implants. There are other visual manoeuvers during surgery, including the use of pins and the assessment of stability, that is also important to assess the length and confirm that biomechanics of the hip are restored appropriately.

Intraoperative imaging is also useful both for direct visualisation of the construct obtained with trials but also to determine leg length directly [76].

Enhanced technology is also helpful. Navigation and robotics have been shown to reduce the incidence of leg length inequality and afford the surgeon a means of avoiding outliers and gaining reproducible outcomes [72, 86, 87].

It is important to check the patient carefully at the end of the procedure. If there is a significant inequality, then it is wisest to address that there and then, rather than wait.

The key to minimising the morbidity and risks of leg length inequality after hip arthroplasty surgery is based around careful patient assessment and planning. We would normally like to put across the learning point that those who fail to plan unfortunately plan to fail.

## Conclusion

Total hip arthroplasty is an effective procedure for the management of end-stage arthritis, characterised by large effect size and results in substantial improvements with respect to the quality of life, symptoms and function. Notwithstanding, early postoperative complications cannot be eliminated, leading to potentially avoidable poor patient outcomes; showcasing the importance of evidence-based prevention strategies. Table 4 summarises the key studies included in this article.

**Table 4:** Summary of key studies included in the manuscript.

Study	Study type	Intervention	Sample size	Findings
Anderson, Dunbar, Murnaghan et al. *Aspirin or Rivaroxaban for VTE Prophylaxis after Hip or Knee Arthroplasty*. N Engl J Med (2018)	Randomised Controlled Trial	Rivaroxaban (10 mg once daily) for 14 days versus the “Wells Protocol” (rivaroxaban 10 mg once daily from up to and including POD 5, plus aspirin 81 mg once daily for 30 days starting on POD 6)	3424	Non-inferiority of the “Wells Protocol”.
				No difference regarding bleeding events.
Gonzalez A, Luime J, Uçkay I, Hannouche D, et al. *Is There an Association Between Smoking Status and Prosthetic Joint Infection After Primary Total Joint Arthroplasty?* J Arthroplasty (2018)	Registry-based cohort study	Pre-operative classification of smoking status into never, former, and current smoker.	8559	1.8 fold increase in the risk of PJI in current and former smokers.
		Incidence rates and hazard ratios (HR) for PJI for different smoking statuses assessed.		
Gibbs VN, McCulloch RA, Dhiman P, et al. *Modifiable risk factors for mortality in revision total hip arthroplasty for periprosthetic fracture.* Bone Jt J (2020)	Retrospective cohort study	Univariate and multivariate logistic regression analyses to identify modifiable factors associated with 90-day and one year mortality in patients undergoing revision THA for periprosthetic hip fracture	203	Dislocation and hospital-acquired pneumonia identified as potentially modifiable risk factors.
Thien T, Chatziagorou G, Garellick G et al. *Periprosthetic femoral fracture within two years after total hip replacement: analysis of 437,629 operations in the nordic arthroplasty register association database.* J Bone Joint Surg Am (2014)	Registry study (Nordic Arthroplasty Register Association database)	Studied seven frequently used stems from 1995 to 2009; two cemented [Exeter and Lubinus SP II] and five uncemented [Bi-Metric, Corail, CLS Spotorno, ABG I, and ABG II])	437,629	2-year incidence of revision 0.47% for uncemented stems and 0.07% for cemented stems.
				Revision increased with age in the uncemented group.
				HR for Exeter stem five times higher than Lubinus SP II stem
				Uncemented stems – ABG II stem increased HR (1.63)
				Corail stem decreased HR (0.47)
Vigdorchik J, Jerabek SA, Mayman DJ et al. *Evaluation of the spine is critical in the workup of recurrent instability after total hip arthroplasty.* Bone Jt J (2019)	Prospective, matched cohort study	Pre-operative imaging – supine and standing AP pelvis and lateral radiographs in patients undergoing revision THA for recurrent instability	222	Utilisation of Hip-Spine Classification System in revision THA resulted in 97% patients having 2-year survival free of dislocation versus 84% without imaging.
Kayani B, Konan S, Thakrar RR et al. *Assuring the long-term total joint arthroplasty.* Bone Joint J (2019)	Prospective cohort study	Compare accuracy in restoration of native centre of rotation in conventional versus robotic THA	75	Robotic THA associated with improved restoration of rotation centre and combined offset.
				More accurate positioning of the acetabular cup within the safe zones with robotic THA.

POD: Post-operative day, PJI: Prosthetic joint infection, HR: Hazard Ratio, AP: anteroposterior, THA: Total Hip Arthroplasty.

Accurate risk stratification to low-intermediate-high individuals should be from the core of any strategy aiming to prevent VTE. A growing body of evidence suggests aspirin is effective in low-risk patients, while tailored prophylaxis and early mobilisation are also key components. PJI can be prevented by early recognising risk factors pre-operatively, optimising the OR environment and wisely choosing the antibiotic regimen.

It is also imperative for the arthroplasty surgeon to take every step to prevent perioperative complications. Periprosthetic fractures are among the most common intra-operative complications of THA, and their frequency can be reduced by employing appropriate prevention strategies. Cementless fixation should be avoided in high-risk individuals such as the elderly with poor bone quality. In addition, the liberal use of prophylactic cerclage and bypassing major stress risers is encouraged. Effective strategies to prevent instability involve the careful choice of articulation based on risk stratification and adopting patient-specific implant positioning.

Attempts to prevent leg-length inequality should be directed at careful patient assessment and pre-and intra-operative planning. The key to minimising the morbidity associated with LLI is to accurately interpret pre-operative radiographs and perform multiple intra-operative tests to ascertain implant position and length. Finally, the experience with navigation and robotics so far has suggested the immense potential of this technology to achieve accurate and reproducible implant positioning.

## Conflict of interest

The authors declare that they have no conflict of interest.

## Funding

There is no funding source.

## Ethical approval

Ethical approval was not required.

## Informed consent

This article does not contain any studies with human participants or animals performed by any of the authors.

## Authors' Contributions

Andreas Fontalis: Conceptualisation, Investigation, Writing – original draft, review & editing.

Daniel J. Berry: Investigation, Writing – original draft, review & editing.

Andrew Shimmin: Investigation, Writing – original draft, review & editing.

Pablo A. Slullitel: Investigation, Writing – original draft, review & editing.

Martin A. Buttaro: Investigation, Writing – original draft, review & editing.

Cao Li: Investigation, Writing – original draft, review & editing.

Henrik Malchau: Investigation, Writing – original draft, review & editing.

Fares S. Haddad: Conceptualisation and planning of the study, Investigation, Writing – original draft, review & editing.
